# Optimal responsiveness and information flow in networks of heterogeneous neurons

**DOI:** 10.1038/s41598-021-96745-2

**Published:** 2021-09-02

**Authors:** Matteo Di Volo, Alain Destexhe

**Affiliations:** 1 Laboratoire de Physique Théorique et Modélisation, Université de Cergy-Pontoise, CNRS, UMR 8089, 95302 Cergy-Pontoise cedex, France; 2 Paris-Saclay University, Institute of Neuroscience, CNRS, Gif sur Yvette, France

**Keywords:** Dynamical systems, Network models

## Abstract

Cerebral cortex is characterized by a strong neuron-to-neuron heterogeneity, but it is unclear what consequences this may have for cortical computations, while most computational models consider networks of identical units. Here, we study network models of spiking neurons endowed with heterogeneity, that we treat independently for excitatory and inhibitory neurons. We find that heterogeneous networks are generally more responsive, with an optimal responsiveness occurring for levels of heterogeneity found experimentally in different published datasets, for both excitatory and inhibitory neurons. To investigate the underlying mechanisms, we introduce a mean-field model of heterogeneous networks. This mean-field model captures optimal responsiveness and suggests that it is related to the stability of the spontaneous asynchronous state. The mean-field model also predicts that new dynamical states can emerge from heterogeneity, a prediction which is confirmed by network simulations. Finally we show that heterogeneous networks maximise the information flow in large-scale networks, through recurrent connections. We conclude that neuronal heterogeneity confers different responsiveness to neural networks, which should be taken into account to investigate their information processing capabilities.

## Introduction

Studying the collective behavior of large numbers of units interacting non-linearly is a classical theme in physical and computational sciences. In biology, such studies are complicated by the fact that the units are usually non identical, but rather display considerable heterogeneity. This particularly apparent in cerebral cortex, where neuronal size and properties are highly heterogeneous^1–12^. Neuronal heterogeneity is particularly high for inhibitory neurons, for which many cell classes were observed^13–16^. Recent works have shown also that heterogeneity in neural response, together with synaptic plasticity, can have a strong impact for response of single neurons as part of a network and can be functional to maintain low firing rates^17–19^. In these cases it is shown that networks of neurons can synchronize even in presence of heterogeneity^20^. In general, in networks of oscillators, as in neuronal networks, such heterogeneity across units (bare frequencies or neurons’ excitability) induces typically desynchronization at a population scale^21–29^. As one would expect, the more the neurons are different the less they are able to synchronize and to correlate their reciprocal activity. Nevertheless, despite this heterogeneity, cortical populations are able to respond coherently to external stimuli and also to generate synchronous collective oscillations^30,31^.

In the present paper, we consider sparse networks of spiking neurons endowed with heterogeneity in neurons’ intrinsic excitability, that we treat independently for excitatory and inhibitory neurons. We investigate the responsiveness of such networks to external inputs, and also compare the heterogeneity to experimental estimates. To yield insights on the causes of the responsiveness properties observed numerically, we employ theoretical methods to derive a mean-field model of heterogeneous networks. We then use the mean-field model to predict the emergence of new activity states due to heterogeneity, a prediction which is tested by numerical simulations of network dynamics. Finally, to determine if the enhanced responsiveness also applies to recurrent connectivity, we considered the propagation of activity in large-scale networks of mean-field models.

## Results

We first investigate the responsiveness of heterogeneous networks of excitatory and inhibitory neurons numerically, and next we use a theoretical approach to understand the mechanisms underlying responsiveness.

### Optimal responsiveness for heterogeneous networks

**Figure 1:**
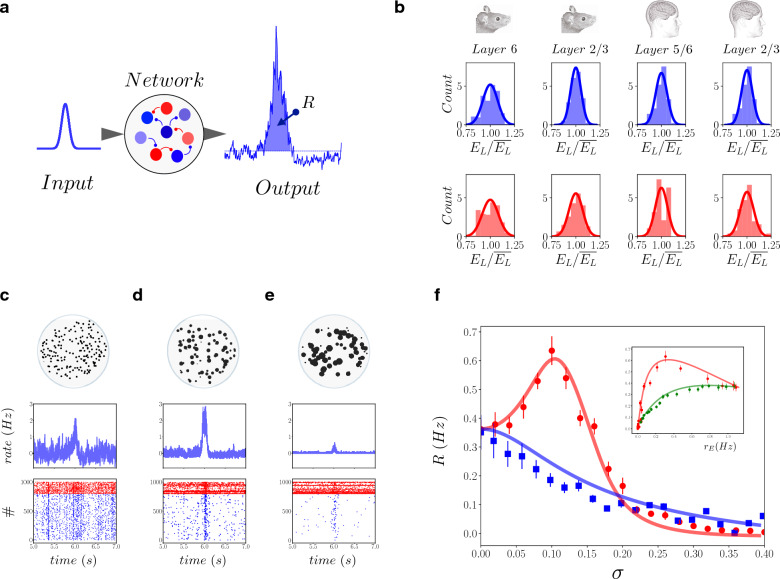
Inhibitory neuron heterogeneity optimizes network responsiveness. (**a**) An afferent excitatory input (Poissonian spike train at a time-dependent frequency $\nu _{ext}(t)$, *Input*) was submitted to a network of excitatory and inhibitory neurons, whose population spiking activity (amount of spikes per time unit, *Output*) is measured. The excess of activity in response to the input (blue area) measures the Responsiveness *R* of the network to the external stimulation. (**b**) Histograms of the resting potential $e_L=E_L/\overline{E_L}$ of excitatoy (inhibitory) neurons in blue (red), top (low) row. Cells originate from the adult human brain, cortical layers 5/6 and 2/3, and mouse cortical layers 6 and 2/3 (Allen Brain Atlas^32^). The continuous line is a Gaussian distribution with the same standard deviation as measured from the data. (**c**) Network activity in response to a time varying input $\nu _{ext}(t)$ of amplitude $A=1$Hz and baseline value $\nu _0=1.5$Hz (see “Methods” section). Excitatory neurons population rate, normalised to pre-stimulus baseline activity, (top row, blue line) and the corresponding raster plot (bottom row), i.e. spiking times of excitatory (inhibitory) neurons marked with blue (red) dots. Panel (**c**) corresponds to an homogeneous network $\sigma _I=0$ , panel (**d**) to $\sigma _I=0.1$ and panel (**e**) to $\sigma _I=0.15$ (in these panels (**c**)–(**e**) $\sigma _E=0$). (**f**) The evoked response *R* is reported in function of the heterogeneity of inhibitory (excitatory) neurons $\sigma =\sigma _I$ ($\sigma =\sigma _E)$, red (blue) dots (squares). Error bars are estimated as the standard deviation over 20 different realisations. Continuous lines report the prediction based on the mean field model (see main text). In the inset, *R* is reported in function of the spontaneous excitatory firing rate $r_E$ without the stimulus (average over 10 s). Red dots are obtained by varying $\sigma _I$ (same data as the main panel). Green dots show *R* in function of $r_E$ for an homogeneous network ($\sigma _I=\sigma _E=0$) and different values of the average resting potential of inhibitory neurons $\overline{E^I_L}$.

We studied networks of sparsely connected excitatory and inhibitory spiking neurons (see “Methods” for details). Each neuron receives afferent excitatory spike trains at a frequency $\nu _{ext}(t)$. To characterise the responsiveness of the network to external stimulation, we considered $\nu _{ext}(t)$ composed by a time constant baseline value $\nu _0$ and a time variation of amplitude *A* (see “Methods”, *Input* in Fig. 1a). The network responds to the stimulation by increasing its population spiking activity (see *Output* in Fig. 1a). The network responsiveness *R* can be estimated as the total amount of evoked spikes by the whole network (see the blue area in the *Output* of Fig. 1a). We compared homogeneous networks to networks constructed from measurements in the adult human and mouse brain from the Allen Brain Atlas^32^. In Fig. 1b, we report the histogram of the resting membrane potential $E_L$ measured experimentally from excitatory (blue, top panels) and inhibitory neurons (red, lower panels) in human and mouse cortical layers. By calling $\overline{E_L}$ the average value of $E_L$, one can see a heterogeneous distribution of the re-scaled resting potential $e_L=E_L/\overline{E_L}$. The distributions have a comparable level of variability, quantified by the standard deviations $\sigma _I$ and $\sigma _E$ (e.g. $\sigma _I\sim 0.07$ and $\sigma _E\sim 0.055$ for Human cortical layers 2/3). Note that heterogeneity in inhibitory cells is generally higher with respect to that in excitatory cells.

To investigate the effect of neuronal heterogeneity, we first studied the effect of excitatory neurons’ heterogeneity $\sigma _E$ and inhibitory neurons’ heterogeneity $\sigma _I$ separately. Panels c,d and e of Fig. 1 show that $\sigma _I$ strongly affects network responsiveness. In these simulations, the network displays spontaneous asynchronous activity where neurons fire irregularly, a dynamical regime due to the balance between excitation and inhibition typically observed in the cortex of awake animals, called asynchronous irregular^17,33,34^. The presence of external excitatory stimuli of short duration (see caption for details) produces a network response (an increase in population spiking activity) corresponding to a transient synchronous event, as it can be observed in the raster plots of Fig. 1c–e. Panel c corresponds to an homogeneous network, while panel d and e correspond to heterogeneous networks with increasing values of $\sigma _I$ ($\sigma _I=0.1$ in panel d and $\sigma _I=0.15$ in panel e, the resting potential $e_L$ follows a Gaussian distribution with fixed average, see “Methods” for details). By increasing $\sigma _I$ we observe a clear increase in the intensity of the burst of response (see panel d), that decreases for larger values of $\sigma _I$ (panel e in Fig 1). This can be quantified by estimating the responsiveness *R* as the amount of evoked spikes by the stimuli (see Fig. 1a and “Methods” section). By looking at Fig. 1f we observe that the same input induces a bell-shaped response *R* in function of the heterogeneity $\sigma _I$, indicating that there is an optimal heterogeneity level ($\sigma _I\sim 0.1$), where the stimuli provokes a strong population response. Eventually, when $\sigma _I$ is too large the response is very weak. On the other side, as it can be noticed from the raster plots in Fig. 1c–e, increasing heterogeneity in inhibitory neurons decreases excitatory neurons spontaneous activity. This is due to the presence of a fraction of inhibitory neurons with high values of the resting potential (closer to the firing threshold) that are more excitable and thus inhibit the excitatory population. In the inset of Fig. 1f we report *R*, as in the main panel, as a function of the excitatory neurons spontaneous activity pre-stimulus $r_E$. We can observe that the responsiveness is maximum, in correspondence of $\sigma _I\sim 0.1$, for a relatively low value of excitatory spontaneous activity (around $r_E\sim 0.3$ Hz). Interestingly, if we consider a homogeneous network ($\sigma _I=0$) and we increase the average resting potential of inhibitory neurons, we observe a decrease of excitatory neurons spontaneous activity pre-stimulus $r_E$ but not an increase of responsiveness *R* (see green dots in the inset of Fig. 1f). This result shows that the increase in the size of the synchronous response is due to the presence of heterogeneity and cannot be replaced by a modification of the average excitability in the corresponding homogeneous network. If we now consider an homogeneous inhibitory population ($\sigma _I=0$), we observe that increasing only the heterogeneity of excitatory neurons $\sigma _E$ has the effect to decrease network responsiveness (blue squares in Fig. 1f). To further investigate the role of heterogeneity in excitatory neurons we study, in the next section, the combined effect of heterogeneity in both populations.

### Responsiveness depends on heterogeneity in excitatory and inhibitory neurons

**Figure 2:**
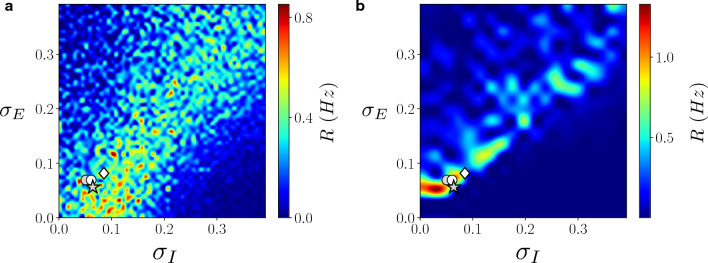
Excitatory and inhibitory heterogeneity determines network responsiveness. (**a**) Responsiveness R as a function of heterogeneity of excitatory ($\sigma _E$) and inhibitory ($\sigma _I$) neurons for networks displaying relatively high levels of spontaneous activity (input baseline $\nu _{0}=1.5\;Hz$ and input amplitude $A=1$ Hz). (**b**) Responsiveness R for networks with a lower level of spontaneous activity (input baseline $\nu _{0}=0.4\;Hz$ and input amplitude $A=0.6$ Hz). Different markers correspond to heterogeneity values estimated from Human cortex layer 2, 3 (white circle), Human cortex layer 5, 6 (grey circle), mouse cortex layer 2/3 (grey star) and mouse cortex layer 6 (white diamond) estimated from Allen Brain database^32^, see Fig. 1.

To explore the combined effect of heterogeneity in both excitatory and inhibitory populations we calculated the responsiveness R as a function of both $\sigma _E$ and $\sigma _I$ (see Fig. 2a). Apart from fluctuations due to the endogenous noise in network dynamics, we observe a region of optimal responsiveness for heterogeneous networks (warm colors in Fig. 2).

Importantly, the experimental values of heterogeneity (Fig. 1b) fall close to the predicted optimal region of network responsiveness (see symbols in Fig. 2a). More specifically, we observe that, if heterogeneity in inhibitory neurons is very high (and thus responsiveness very low), increasing heterogeneity in excitatory neurons permits to increase responsiveness R. In another way, both heterogeneity cooperate to increase network responsiveness *R*, that is low for homogeneous or too heterogeneous networks. Thus, the model predicts that the optimal heterogeneity matches that found in real neural networks, suggesting that this aspect is crucial to understand their responsiveness. In order to study the generality of this scenario, we consider a very different setup, with a lower background excitatory afferent input to the network (see caption in Fig. 2). In this parameter setup the homogeneous network has a lower spontaneous activity. In Fig. 2b we report the responsiveness *R* for this setup. We observe indeed that also in this case an heterogeneous network yields a higher responsiveness *R* and that the experimental amount of heterogeneity falls in the region of high responsiveness *R*. This result shows that, also across different parameter setups (other parameters are also studied in next sections), an heterogeneous network corresponds to an optimal responsiveness. Moreover, the optimal responsiveness appears for values of heterogeneity close to experimental amounts. In order to test the robustness of this observation, we have verified that changing network realization and initial conditions the results are the same (see Supplementary Information).

### Optimal responsiveness comes from pushing the network at the edge of a dynamical transition

To determine the mechanism at the origin of the enhanced responsiveness due to neuronal heterogeneity, we developed a mean-field approach explicitly taking into account diversity. We started from a mean-field model previously introduced for homogeneous neural populations^35–37^. We extend this approach to heterogeneous systems by employing a technique, called heterogeneous mean field (HMF), successfully applied previously to model networks with heterogeneous connectivity^38,39^ and extended here to networks with heterogeneous cell properties (see “Methods” section). This procedure describes the collective dynamics of large sparsely connected networks of heterogeneous neurons with a relatively simple three dimensional model. This HMF model predicts the time evolution of excitatory neurons population firing rate $r_E(t)$ and of inhibitory neurons population firing rate $r_I(t)$. By comparing the prediction of the HMF model to estimations from direct numerical simulations of a large network of neurons, we observed a very good agreement, that can be appreciated from the prediction of the response *R* in function of $\sigma _I$($\sigma _E$) (see continuous lines in Fig. 1d).

**Figure 3:**
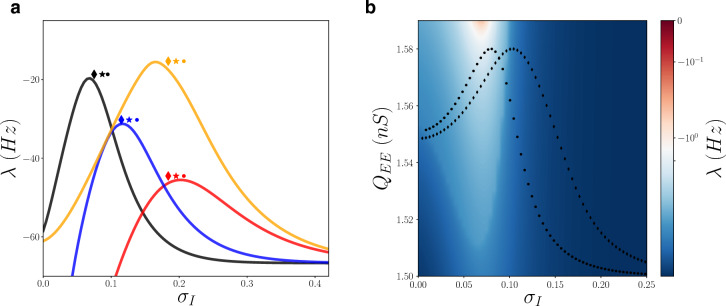
Enhanced responsiveness corresponds to regions closer to instability. (**a**) The second largest stability Lyapunov exponent $\lambda$ (real part) of asynchronous dynamics as a function of the heterogeneity of inhibitory neurons ($\sigma _I$). Different colors indicate different parameters of the baseline external drive and the strength of excitatory-excitatory quantal conductance ($\nu _{0},Q_{EE}$), i.e. black (1.5 Hz, 1.5 nS), red (3 Hz, 1.5 nS), blue (2 Hz, 1.5 nS) and orange (3 Hz, 1.65 nS). Symbols are located at the value of $\sigma _I$ for which the responsiveness *R* is maximum (same color code as continuous line). Different symbols indicate different amplitudes *A* of the input, diamond ($A=0.1$ Hz), star ($A=0.5$ Hz) and dot ($A=1$ Hz). (**b**) The second largest stability Lyapunov exponent $\lambda$ of asynchronous dynamics as a function of the heterogeneity of inhibitory neurons ($\sigma _I$) and the strength of excitatory-excitatory quantal conductance $Q_{EE}$ for a baseline external drive $\nu _{0}=1.5$ Hz. The dotted (diamond) line is the responsiveness *R* for an input amplitude $A=0.1$ Hz ($A=1\;Hz$) and $Q_{EE}=1.5$ nS (as in Figs. 1 and 2). Such responsiveness has been properly rescaled on the y-axes (i.e. multiplied by an ad-hoc factor) in order to fit in the image.

The excellent match of the continuous curves in Fig. 1 shows that the HMF correctly predicts the numerical observations of an optimal amount of heterogeneity in inhibitory neurons for population response. We can go further, and exploit the simplicity of the HMF model to understand what are the differences between more or less responsive asynchronous states. By computing the stationary solution from the HMF we estimate the relative stability eigenvalues $\{\lambda _i\}$. The real part of the second largest exponent $\lambda$ is reported in Fig. 3a for different parameter setups. The closer $\lambda$ to the critical value $\lambda _c=0$ the less the asynchronous state is stable to perturbations. We observe that, independently of model parameters it exists an intermediate value of heterogeneity $\sigma _I$ for which $\lambda$ is maximum and closer to the transition point $\lambda =0$. We have then estimated the responsiveness *R* for these setups and we observe that the maximum responsiveness appears at values of the heterogeneity $\sigma _I$ at which the asynchronous state is less stable (see symbols in Fig. 3a). In Fig. 3b then we report $\lambda$ in function of $\sigma _I$ and the intensity of excitatory–excitatory neurons synaptic strength $Q_{EE}$. We observe that, increasing $Q_{EE}$ there exists a region where the asynchronous state becomes marginally stable (red region, $\lambda$ close to zero). By superimposing the responsiveness *R* from Fig. 1d (dotted line, in this case for fixed $Q_{EE}=1.5$ nS), we can see that the value of $\sigma _I$ for which the responsiveness is maximum corresponds to a network that is closer to the transition point where the asynchronous regime becomes unstable.

### Heterogeneity can induce new dynamical regimes

**Figure 4:**
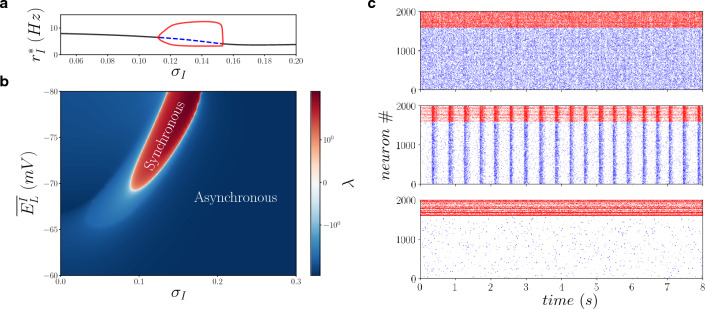
Heterogeneous networks admit more dynamical regimes compared to homogeneous networks. (**a**) Inhibitory neurons population stationary firing rate $r^*_I$ in function of $\sigma _I$ for $\overline{E^I_L}=-70$ mV. Black line indicates stable ($\lambda <0$) asynchronous state, dashed blue line indicates unstable ($\lambda >0$) asynchronous state where a limit cycle appears (red lines indicates maximum and minimum value of $r_I$ in time). (**b**) The second stability Lyapunov exponent $\lambda$ (real part) of the asynchronous state as a function of the average resting potential of inhibitory neurons $\overline{E^I_L}$ and their standard deviation $\sigma _I$. Whenever $\lambda >0$ the asynchronous state is unstable and a stable limit cycle appears. In direct network simulations we observe sparsely synchronous oscillations (see raster plots in panel (**c**) where we use $\overline{E^I_L}=-70$ mV and $\sigma _I=0,0.12,0.2$ from top to bottom). In these simulations $Q_{EE}=1.53$ nS.

Finally, to determine what the unstable regions correspond to, we investigated heterogeneous networks both numerically and theoretically using the mean-field model. Figure 4a shows that, for intermediate amount of heterogeneity the asynchronous state can be unstable (blue dashed line in panel a). This dynamical phase corresponds to an oscillatory regime at the level of the whole network, arising from a super-critical Hopf bifurcation where the amplitude of the limit cycle (red lines in panel a) smoothly increases in function $\sigma _I$. In panel b of Fig. 4 we observe that this oscillating synchronous regime (present whenever the asynchronous state is unstable, $\lambda >0$) appears only for heterogeneous networks ($\sigma _I$>0) but the location of the synchronous region depends on the average value of neurons’ resting potential $\overline{E^I_L}$ (notice that in panel a of Fig. 4 we used $\overline{E^I_L}=-70$ mV and in Figs. 1, 2 and 3 we used $\overline{E^I_L}=-65$ mV ). In order to verify these theoretical predictions, in panel c we finally perform numerical simulation of the neuronal network where we observe that the limit cycle corresponds indeed to collective network oscillations (middle panel) that disappear in homogeneous or too heterogeneous setups (higher and lower panels). This shows that not only heterogeneity can induce higher responsiveness, but it can also induce new dynamical regimes. The dynamics of heterogeneous networks is therefore richer.

### Heterogeneity boosts information flow in large-scale networks

**Figure 5:**
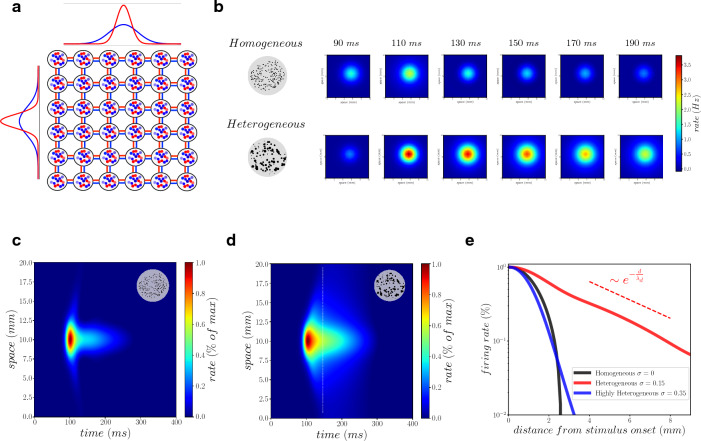
Enhanced activity propagation in large-scale heterogeneous networks. (**a**) Two dimensional lattice of connected mean-field models. The connectivity between excitatory (inhibitory) cells is drawn from a Gaussian distribution with standard deviation of length $l_{exc}=2$ mm ($l_{inh}=1$ mm), see blue (red) curves. (**b**) Response of the system (firing rate of excitatory neurons) following a Gaussian afferent stimulus (see “Methods”). The upper row stands for locally homogeneous networks ($\sigma _I=0$) while lower row for locally heterogeneous networks ($\sigma _I=0.15$). (**c**) Spatio–temporal profile of excitatory neurons firing rate normalised by its maximum in space and time ($\sigma _I=0$). (**d**) Same as (**c**) but for $\sigma _I=0.15$. (**e**) Normalised firing rate in function of the distance *d* from stimulus onset at a specific time [see dashed white line in panel (**d**)]. Different colors stand for different levels of heterogeneity (see the legend inside the panel). The dashed red line shows a fit of the exponential decay of activity found in the heterogeneous system (red line, $\sigma _I=0.15$), with $\lambda _d \sim 3.5$ mm.

To determine if the enhanced responsiveness applies not only to external inputs but also to the flow of information through recurrent connectivity, we considered large-scale networks where each unit is a population of heterogeneous neurons. We considered a two dimensional lattice of mean-field models, that are interconnected to each other via Gaussian connectivity profiles (see Fig.  5a and “Methods” section). According to anatomical connectivity estimates, inhibitory neurons have a short-range connectivity (red curves in Fig. 5a) at variance with excitatory connectivity (blue curves in Fig. 5a)^40^. Importantly, we integrate distance-dependent propagation delays due to the finite velocity of axonal conduction of action potentials (we considered here an axonal conduction velocity of 0.3 m/s, see “Methods” section).

We stimulated this large-scale model by an external input (see “Methods” section) and we compared a model with locally heterogeneous neurons (each node is modeled with an heterogeneous mean field with $\sigma _I=0.15$) versus the same model with locally homogeneous neurons (each node is modeled with an homogeneous mean field with $\sigma _I=0$). As it can be appreciated from Fig. 5b, both the heterogeneous and the homogeneous model respond with an activation that propagates in space as a wave. We observed that the intensity of the wave is much larger in the model with locally heterogeneous networks. This is a direct consequence of the enhanced responsiveness of each node. But does local heterogeneity also impacts the spatial extent of the wave through recurrent connections? To answer this question we compared the homogeneous and the heterogeneous system by normalising the firing rate activity with its maximum in space and time, in order to highlight the spatio-temporal profile of the response. As it can be observed from panels c and d in Fig. 5, the homogeneous and the heterogeneous systems have different spatio-temporal profiles, indicating that local heterogeneity influences not only the intensity of the response to the stimulus, but also its spatio-temporal pattern through recurrent interactions. Remarkably the heterogeneous system has much longer-range spatial propagation of activity. More specifically, for an optimal amount of heterogeneity we observed a much enhanced propagation of the activity through the network (red curve in Fig. 5e), compared with homogeneous and highly heterogeneous networks.

These results show that an optimal amount of local heterogeneity has a double effect at large scale. First, it amplifies the intensity of the response to the external input (Fig. 5b, a consequence of local enhanced responsiveness) and, second, enhanced responsiveness also applies to recurrent inputs, which results in a long range propagation of activity across the network (Fig. 5e).

## Discussion

In conclusion, we report here four findings. First, we have found that the heterogeneity of inhibitory neurons, which has been well documented experimentally^13–16^, optimises the responsiveness of spontaneously active networks to external stimuli. There appears a resonance peak as a function of the level of heterogeneity. We have here studied the heterogeneity in neurons’ resting potential, that is experimentally quantified (see Fig. 1b and Supplementary Information) and determines neurons’ proximity to firing threshold (and thus neurons’ excitability). On the other side our results do not limit to only this cellular parameter. We have indeed found, both theoretically and by performing network simulations, that an optimal responsiveness appears also for intermediate heterogeneity in neurons’ leakage conductance, while almost no effect is observed for heterogeneity in neurons’ membrane capacitance (see Supplementary Information).

A similar effect of diversity-induced resonance was previously observed in excitable or bistable systems^41–45^, where heterogeneity creates active excitation clusters which were absent in the quiescent homogeneous system. We showed here a different type of heterogeneity, that of inhibitory neurons, can induce optimal population responses. More generally, we found that the heterogeneity of both excitatory and inhibitory populations induces optimal responsiveness in spontaneously active sparse networks with irregular firing activity. Importantly, we found that the level of heterogeneity measured experimentally across different cortical layers and species (Human and Mouse) corresponds to the resonance peak, which suggests that cortical networks may have naturally evolved towards optimal responsiveness by adjusting their heterogeneity. Moreover, while several studies reported that heterogeneity can enhance coding in uncoupled networks^46,47^ and decrease neuronal correlations^48–50^, we report here that a higher input–output population response is linked to an increased tendency to synchronization in heterogeneous networks. The coding capabilities of neural networks will therefore be largely affected by neuronal heterogeneity, which opens interesting perspectives for future studies.

Second, we found that the enhanced responsiveness of heterogeneous networks is paralleled with a decreased stability of the spontaneous activity regime. To obtain this result, we designed a mean-field model that explicitly includes heterogeneity, and which can capture this diversity-induced resonance. This new mean-field formulation keeps track of microscopic complexity, compared to traditional mean-field approaches which implicitly assume homogeneous systems and would not predict the correct responsiveness. This also shows that responsiveness must be understood using the knowledge of the spontaneous activity of the network—and in particular its level of stability. The relation between instability and responsiveness also constitutes a promising subject to further explore in the future.

In this work we have studied heterogeneity in the intrinsic proprieties of neurons, but it is known that heterogenity can be found also in the structure of connections (say for example in neurons’ in-degrees). Structural Heterogeenity (i.e. in the topology of connections) can have a strong impact in network dynamics^11,51–53^. Our mean field approach is general and can be employed in the future to study the impact of heterogeneity in neurons’ connectivity.

Third, we have shown that neuronal heterogeneity is not only important for responsiveness, but also can induce new dynamical regimes. Using the mean-field models, we could predict a transition to a sparsely synchronous collective oscillation regime, which was confirmed by network simulations. This type of diversity-induced oscillations reminds some aspects found in noise-induced transitions in dynamical systems^54,55^. Whether the effects of heterogeneity could be considered as analogous to the effect of noise (“quenched noise”) in neural networks is also an interesting direction for future studies.

Finally, we have found that the enhanced responsiveness enables activity to propagate much easier in large-scale heterogeneous networks. The enhanced response not only applies to the stimulus, but also it applies to information flow through recurrent excitatory inputs. As a result, comparing heterogeneous to homogeneous networks, a given stimulus produces a larger local response, and in addition, this response also propagates to a larger spatial extent because the effect of recurrent excitatory inputs is also amplified by heterogeneity. Thus, we conclude that heterogeneous networks can provide activity propagation at a level much superior compared to a homogeneous system, and thus will necessarily better propagate information and make it available to larger brain areas.

## Methods

### Network model

We examined networks of excitatory and inhibitory neurons connected through conductance based synapses. We used networks of $N=10,000$ neurons, 80% of excitatory ($N_E=0.8\;N$) and 20% of inhibitory ($N_I=0.27\;N$) neurons. The membrane potential $V_i$ of each neuron evolves according to the Adaptive Exponential integrate and fire model (AdExp)^56^:

1 $$\begin{aligned}&C_{m}\dot{V}_i = g_L(E_L^i-V_i)+g_Le^{\frac{V_i-v_{th}}{\Delta }}+I_{s}^i-w_i \end{aligned}$$

2 $$\begin{aligned}&\tau _w\dot{w}_i =-w_i + b\sum _{\{ t_i^ {sp}\}} \delta (t- t_i^ {sp}), \end{aligned}$$

where $C_m=200$ pF is the membrane capacitance, $g_L = 15$ nS the leakage conductance, $v_{th}=50$ mV the effective threshold and $\Delta$ defines the action potential rise ($\Delta =0.5$ mV for inhibitory neurons and $\Delta =2$ mV for excitatory neurons). The adaptation current $w_i$ increases of an amount $b=60$ nS at each spike emitted by neuron *i* at times $\{ t_i^ {sp}\}$ and has an exponential decay with time scale $\tau _w=500$ ms. Only excitatory neurons have spike frequency adaptation, while for inhibitory neurons $b=0$. The current $I_{s}^i$ is the current received by neuron *i* from other neurons in the network. We consider a random graph where each couple of neurons is connected with probability $p=0.05$. By calling $\{ t_j^ {sp}\}$ the ensemble of spiking times of neuron *j* we have, for an excitatory post-synaptic neuron *i*:

3 $$\begin{aligned}&I_{s}^i= g_i^{EE}(V_i-E_E)+g_i^{EI}(V_i-E_I) \end{aligned}$$

4 $$\begin{aligned}&\tau _{s}\dot{g}_i^{EE}=-g_i^{EE}+Q_{EE}\sum _{\{ t_j^ {sp}\}\in (E)}\delta (t- t_j^ {sp}) \end{aligned}$$

5 $$\begin{aligned}&\tau _{s}\dot{g}_i^{EI}=-g_i^{EI}+Q_{EI}\sum _{\{ t_j^ {sp}\}\in (I)}\delta (t- t_j^ {sp}), \end{aligned}$$

where $E_{E,I}$ is the reversal for excitatory ($E_{E}=0$ mV) and inhibitory synapses ($E_{I}=-80$ mV), $\tau _{s}=5$ ms the synaptic decay time and $Q_{EE}$ ($Q_{EI}$) is the interaction strength of excitatory (inhibitory) synapses to excitatory neurons. The same equations (with $g_i^{EE}\rightarrow g_i^{IE}$, $g_i^{EI}\rightarrow g_i^{II}$, $Q_{EE}\rightarrow Q_{IE}$ and $Q_{EI}\rightarrow Q_{II}$ ) apply for inhibitory post-synaptic neurons. We fix $Q_{EI}=Q_{II}=5$ nS and $Q_{EE}=Q_{IE}=1.5$ nS (we employed different values of $Q_{EE}$ in Fig. 3 and in Fig. 4, see the relative caption).

Each neuron receives an external Poissonian train of excitatory spikes at a rate $\nu _{ext}$. The value of $\nu _{ext}$ determines the amount of ongoing spontaneous activity in the network. In order to study the response to external stimuli we considered a time varying $\nu _{ext}(t)$ of the form $\nu _{ext}(t)=\nu _0+Ae^{-\frac{(t-t_0)^2}{2T^2}}$, where *A* is the input amplitude, $t_0$ the time when input is maximum and $T=50$ ms measures the duration of the input. We have employed $\nu _0=1.5$ Hz in all the numerical simulations, apart from Figs. 2b and 3 (see Figure caption for details). In Fig. 1 we employed $t_0=6$ s, $\nu _0=1.5$ Hz and $A=1$ Hz. The responsiveness *R* is estimated by computing the amount of spikes of excitatory neurons while the input is on (i.e. between $t=t_0-3T$ and $t=t_0-3T$) minus the baseline activity (average of excitatory spike rate for $A=0$). Responsiveness *R* is estimated by averaging over 20 different repetitions of this procedure.

To model heterogeneity, we considered a Gaussian distribution of the resting potential $E_L^i$ of inhibitory (excitatory) population ${\mathcal {N}}(\overline{E^{E,I}_L},\,\overset{\sim }{\sigma }_{E,I}^{2})$ with average $\overline{E^{E,I}_L}$ and standard deviation $\overset{\sim }{\sigma }_{E,I}$. We considered $\overline{E_L^{E}}=\overline{E_L^{I}}=-65$ mV if not stated otherwise (e.g. we used different values of $\overline{E_L^{I}}$ in the inset of Fig. 1d and in Fig. 4). The re-scaled standard deviation $\sigma _{E,I}=\overset{\sim }{\sigma }_{E,I}/\overline{E^{E,I}_L}$ is the main parameter to quantify heterogeneity. The same definitions apply for heterogeneity in other parameters (see Supplementary Information for $g_L$ and $C_m$).

### Mean field model

In the homogeneous case a mean field model for this network has been recently developed^37^. By employing a Markovian approximation over a time scale $\tau =15$ ms and by considering a sufficiently slow time scale for the dynamics of adaptation $\tau _w$, mean field equations read:

6 $$\begin{aligned}&\tau \dot{r}_I = F^I(r_E+\nu _{ext},r_I)-r_I \end{aligned}$$

7 $$\begin{aligned}&\tau \dot{r}_E = F^E(r_E+\nu _{ext},r_I,W)-r_E \end{aligned}$$

8 $$\begin{aligned}&\tau _w\dot{W}=-W+br_E, \end{aligned}$$

where $r_E$ ($r_I$) is excitatory (inhibitory) neurons population firing rate, *W* is excitatory neurons average spike frequency adaptation, $F^I(\nu _{E},\nu _{I})$ and $F^E(\nu _{E},\nu _{I},W)$ are the transfer functions of inhibitory and excitatory neurons, respectively. They measure the stationary firing rate of one neuron when receiving an excitatory (inhibitory) Poissonian spike train at a rate $\nu _{E}$ ($\nu _{I}$).

In the case of heterogeneous inhibitory neurons, a parameter *x*, say neurons reversal potential $E_L$, is distributed according to a probability density function *P*(*x*). In the limit of large networks we can decompose inhibitory neurons in classes, each one characterized by a parameter *x* and by its own transfer function $F_x^I(r_E+\nu _{ext},r_I)$. We indicate with $r_x^I$ the firing rate of the class of neurons with parameter *x*. The whole population rate is $r_I=\int dxP(x)r_x^I$. The equations are closed by a self consistency equation for the mean input received by one neuron and we need to replace Eq. (6) with

9 $$\begin{aligned}&\tau \dot{r}_I = \int dxP(x)F^I_{x}(r_E+\nu _{ext},r_I)-r_I. \end{aligned}$$

Notice that the model still stays three dimensional but keeps track of the distribution *P*(*x*) of heterogeneity. In the case $P(x)=\delta (x-x_0)$ we recover the homogeneous model.

The same procedure applies for excitatory neurons. Nevertheless, in this case each class *x* is characterised by its own adaptation variable $w_x$. For each class with parameter *x* we get:

10 $$\begin{aligned}&\tau \dot{r}^E_x = F^E_x(r_E+\nu _{ext},r_I,w_x)-r_E \end{aligned}$$

11 $$\begin{aligned}&\tau _w\dot{w}_x=-w_x+br^E_x. \end{aligned}$$

In this case the population quantities can be written as $W=\int P(x)w_x$ and $r_E=\int P(x)r^E_x$. These equations can be solved by sampling *P*(*x*) and we found that a sampling of around 50 points gives an accurate precision. In our work we employ the mean field for heterogeneous excitatory neurons only in Fig. 1d, for which we employed a sampling of 50 points.

Nevertheless, it is possible to reduce the dimensionality of this model by making the hypothesis that $w_x$ is slow enough. The stationary solution is $w_x=br^E_x$. In order to follow the dynamics of adaptation we evolve the population variable *W* as $\tau _w\dot{W}=-W+br^E$ and estimate the adaptation of each class from the equation $w_x=Wr^E_x/r_E=(W/r_E)F^E_x(r_E+\nu _{ext},r_I,w_x)$. In this way the model stays three dimensional. Preliminary results indicate that this is a good approximation for the population dynamics in the heterogeneous case.

Finally, the estimation of neuron transfer functions for the AdExp model $F^{E/I}(r_E,r_I,W)$ is done through a semi-analytical fitting procedure^37^ (see Supplementary Information).

### Spatially extended model

To model large-scale networks, we considered a square lattice of length $L=20$ mm composed of $M \times M$ nodes, we employed M = 100 in the simulations of Fig. 5. Each node is modeled as a mean field model. In the limit of large *M* the dynamics of each node at a location (*x*, *y*) follows:

12 $$\begin{aligned}&\tau \frac{\partial r_E(x,y)}{\partial t} =-r_E(x,y)+F^E(\nu _E(x,y)+\nu _{inp}(x,y,t),\nu _I(x,y),W(x,y)) \end{aligned}$$

13 $$\begin{aligned}&\tau \frac{\partial r_I(x,y)}{\partial t} =-r_I(x,y)+ \int dzP(z)F^I_{z}(\nu _E(x,y)+\nu _{inp}(x,y,t),\nu _I(x,y)) \end{aligned}$$

14 $$\begin{aligned}&\tau _w\dot{W}(x,y)=-W(x,y)+br_E(x,y), \end{aligned}$$

where *P*(*z*) is the distribution of heterogeneity, i.e. a Gaussian distribution with average $\overline{E^{I}_L}$ and rescaled standard deviation $\sigma _I$, and $\nu _E(x,y)$ ($\nu _I(x,y)$) is the excitatory (inhibitory) input incoming in (*x*, *y*) from the other lattice locations, i.e.

15 $$\begin{aligned}&\nu _E(x,y)=\int dx_1 \int dy_1 G_E(x-x_1,y-y_1)r_E\left(x_1,y_1,t-\frac{d}{v_c}\right) \end{aligned}$$

16 $$\begin{aligned}&\nu _I(x,y)=\int dx_1 \int d_y1 G_I(x-x_1,y-y_1)r_I\left(x_1,y_1,t-\frac{d}{v_c}\right), \end{aligned}$$

where $d=\sqrt{(x-x_1)^2+(y-y_1)^2}$ is the distance between two points in the lattice and $G_E$ ($G_I$) is the excitatory (inhibitory) connectivity in space. We consider a Gaussian connectivity in both directions with standard deviation $\Delta _E=2$ mm ($\Delta _I=1$ mm) for excitatory (inhibitory) connections. We considered an axonal conduction velocity $v_c=0.3$ m/s. We considered an external stimulation as a stationary input $\nu _{inp}(x,y,t)$:

17 $$\begin{aligned} \nu _{inp}(x,y,t)=\frac{A}{2\pi \Delta _{inp}^2}e^{-\frac{(t-t_0)^2}{2T^2}}e^{-\frac{(x-x_0)^2}{2\Delta _{inp}^2}}e^{-\frac{(y-y_0)^2}{2\Delta _{inp}^2}}, \end{aligned}$$

with $T=50$ ms, $\Delta _{inp}=1.5\;mm$, $A=1\;Hz$, $t_0=100$ ms and $x_0=y_0=10$ mm.

### Supplementary Information

Supplementary Information.
