# Author Correction: Optimal responsiveness and information flow in networks of heterogeneous neurons

**DOI:** 10.1038/s41598-024-59898-4

**Published:** 2024-04-29

**Authors:** Matteo Di Volo, Alain Destexhe

**Affiliations:** 1 Laboratoire de Physique Théorique et Modélisation, Université de Cergy-Pontoise, CNRS, UMR 8089, 95302 Cergy-Pontoise cedex, France; 2 Paris-Saclay University, Institute of Neuroscience, CNRS, Gif sur Yvette, France

Correction to: *Scientific Reports* 10.1038/s41598-021-96745-2, published online 02 September 2021

The original version of this article contained an error in the legends of Figure 3 and Figure 4, where it was erroneously stated that the Lyapunov exponent plotted in Figure 3 and Figure 4b is the largest Lyupanov exponent.

In Figure 3,

“Enhanced responsiveness corresponds to regions closer to instability. (**a**) The largest stability Lyapunov exponent $\lambda$ (real part) of asynchronous dynamics as a function of the heterogeneity of inhibitory neurons (${\sigma }_{I}$). Different colors indicate different parameters of the baseline external drive and the strength of excitatory-excitatory quantal conductance (${\nu }_{0},{Q}_{EE}$), i.e. black (1.5 Hz, 1.5 nS), red (3 Hz, 1.5 nS), blue (2 Hz, 1.5 nS) and orange (3 Hz, 1.65 nS). Symbols are located at the value of ${\sigma }_{I}$ for which the responsiveness *R* is maximum (same color code as continuous line). Different symbols indicate different amplitudes *A* of the input, diamond ($A=0.1$ Hz), star ($A=0.5$ Hz) and dot ($A=1$ Hz). (**b**) The larger stability Lyapunov exponent $\lambda$ of asynchronous dynamics as a function of the heterogeneity of inhibitory neurons (${\sigma }_{I}$) and the strength of excitatory-excitatory quantal conductance ${Q}_{EE}$ for a baseline external drive ${\nu }_{0}=1.5$ Hz. The dotted (diamond) line is the responsiveness *R* for an input amplitude $A=0.1$ Hz ($A=1 Hz$) and ${Q}_{EE}=1.5$ nS (as in Figs. 1 and 2). Such responsiveness has been properly rescaled on the y-axes (i.e. multiplied by an ad-hoc factor) in order to fit in the image.”

now reads:

“Enhanced responsiveness corresponds to regions closer to instability. (**a**) The second largest stability Lyapunov exponent $\lambda$ (real part) of asynchronous dynamics as a function of the heterogeneity of inhibitory neurons (${\sigma }_{I}$). Different colors indicate different parameters of the baseline external drive and the strength of excitatory-excitatory quantal conductance (${\nu }_{0},{Q}_{EE}$), i.e. black (1.5 Hz, 1.5 nS), red (3 Hz, 1.5 nS), blue (2 Hz, 1.5 nS) and orange (3 Hz, 1.65 nS). Symbols are located at the value of ${\sigma }_{I}$ for which the responsiveness *R* is maximum (same color code as continuous line). Different symbols indicate different amplitudes *A* of the input, diamond ($A=0.1$ Hz), star ($A=0.5$ Hz) and dot ($A=1$ Hz). (**b**) The second largest stability Lyapunov exponent $\lambda$ of asynchronous dynamics as a function of the heterogeneity of inhibitory neurons (${\sigma }_{I}$) and the strength of excitatory-excitatory quantal conductance ${Q}_{EE}$ for a baseline external drive ${\nu }_{0}=1.5$ Hz. The dotted (diamond) line is the responsiveness *R* for an input amplitude $A=0.1$ Hz ($A=1 Hz$) and ${Q}_{EE}=1.5$ nS (as in Figs. 1 and 2). Such responsiveness has been properly rescaled on the y-axes (i.e. multiplied by an ad-hoc factor) in order to fit in the image.”

In Figure 4,

“Heterogeneous networks admit more dynamical regimes compared to homogeneous networks. (**a**) Inhibitory neurons population stationary firing rate ${r}_{I}^{*}$ in function of ${\sigma }_{I}$ for $\overline{{E_{L}^{I} }} = - 70$ mV. Black line indicates stable ($\lambda <0$) asynchronous state, dashed blue line indicates unstable ($\lambda >0$) asynchronous state where a limit cycle appears (red lines indicates maximum and minimum value of ${r}_{I}$ in time). (**b**) The largest stability Lyapunov exponent $\lambda$ (real part) of the asynchronous state as a function of the average resting potential of inhibitory neurons $\overline{{E_{L}^{I} }}$ and their standard deviation ${\sigma }_{I}$. Whenever $\lambda >0$ the asynchronous state is unstable and a stable limit cycle appears. In direct network simulations we observe sparsely synchronous oscillations (see raster plots in panel (**c**) where we use $\overline{{E_{L}^{I} }} = - 70$ mV and ${\sigma }_{I}=\mathrm{0,0.12}, 0.2$ from top to bottom). In these simulations ${Q}_{EE}=1.53$ nS.”

now reads:

“Heterogeneous networks admit more dynamical regimes compared to homogeneous networks. (**a**) Inhibitory neurons population stationary firing rate ${r}_{I}^{*}$ in function of ${\sigma }_{I}$ for $\overline{{E_{L}^{I} }} = - 70$ mV. Black line indicates stable ($\lambda <0$) asynchronous state, dashed blue line indicates unstable ($\lambda >0$) asynchronous state where a limit cycle appears (red lines indicates maximum and minimum value of ${r}_{I}$ in time). (**b**) The second largest stability Lyapunov exponent $\lambda$ (real part) of the asynchronous state as a function of the average resting potential of inhibitory neurons $\overline{{E_{L}^{I} }}$ and their standard deviation ${\sigma }_{I}$. Whenever $\lambda >0$ the asynchronous state is unstable and a stable limit cycle appears. In direct network simulations we observe sparsely synchronous oscillations (see raster plots in panel (**c**) where we use $\overline{{E_{L}^{I} }} = - 70$ mV and ${\sigma }_{I}=\mathrm{0,0.12,0.2}$ from top to bottom). In these simulations ${Q}_{EE}=1.53$ nS.”

As a result, in the Results section, under the subheading ‘Optimal responsiveness comes from pushing the network at the edge of a dynamical transition’,

“The maximum exponent $\lambda$ is reported in Fig. 3a for different parameter setups.”

now reads:

“The real part of the second largest exponent  $\lambda$ is reported in Fig. 3a for different parameter setups.”

The original article has been corrected.

