# Supplementary material for: Optimal responsiveness and information flow in networks of heterogeneous neurons
Source: Sci Rep. 2021 Sep 2;11:17611. doi: 10.1038/s41598-021-96745-2 (PMC8413388; doi:10.1038/s41598-021-96745-2)
Supplement: Supplementary file 1 — Supplementary Information. [file 41598_2021_96745_MOESM1_ESM.pdf]

Supplemetary Material for "Optimal responsiveness and information flow in networks of heterogeneous neurons"

Matteo Di Volo[1,*] **and Alain Destexhe**[2]

[1]Laboratoire de Physique Théorique et Modélisation, Université de Cergy-Pontoise, CNRS, UMR 8089, 95302 Cergy-Pontoise cedex, France
[2]Paris-Saclay University, Institute of Neuroscience, CNRS, Gif sur Yvette, France
[*]matteo.di-volo@u-cergy.fr
July 13, 2021

We describe here supplementary information for different aspects developed in the main manuscript.

1 Responsiveness for different parameters of heterogeneity

We have investigated the responsiveness of the network for heterogeneity for other cellular parameters than the resting level or leakage reversal potential (σ_{E_L} as in Fig.1d of the main article). We report here the results for the membrane capacitance C_m and the leakage conductance g_L. We considered a Gaussian distribution of g_L (C_m) across inhibitory neurons.

The re-scaled standard deviation $\sigma_{g_L}(\sigma_{C_m})$, i.e. the standard deviation of the distribution divided by the mean, quantifies the amount of heterogeneity. We observe in Fig.1 that an optimal responsiveness in function of heterogeneity is not specific to the only E_L but it appears (even if shifted to higher heterogeneity values) also for the leakage conductance g_L. On the contrary, heterogeneity in membrane capacitance seems to have a small effect on network responsiveness (panel c).

2 Transfer function calculation

Estimation of neuron transfer functions

In the case of the conductance based Adaptive Exponential Integrate and Fire model there is not an analytic way to evaluate the transfer function. We perform a semi-analytical derivation of the transfer function F of excitatory and inhibitory neurons[1]. The method is based on the hypothesis that the output firing rate of a neuron can be written as a function of the statistics of its sub-threshold voltage dynamics, i.e. the average sub-threshold voltage μ_V, its standard deviation σ_V and its time correlation decay time τ_V.

From input rates to sub-threshold voltage moments

The mean membrane potential is obtained by taking the stationary solution to static conductance given by the mean synaptic bombardment with firing rates (ν_E, ν_I)[2]. We can calculate the average $\mu_{Ge,Gi}$ and standard deviation $\sigma_{Ge,Gi}$ of such bombardment for both excitatory and inhibitory process in the case spikes follow a Poissonian statistics:

$$
\begin{aligned}
&\mu_{Ge}(\nu_E,\nu_I) = \nu_E K_E \tau_E Q_E \\
&\sigma_{Ge}(\nu_E,\nu_I) = \sqrt{\frac{\nu_E K_I \tau_e}{2}} Q_E \\
&\mu_{Gi}(\nu_E,\nu_I) = \nu_I K_I \tau_I Q_I \\
&\sigma_{Gi}(\nu_E,\nu_I) = \sqrt{\frac{\nu_I K_I \tau_i}{2}} Q_I,
\end{aligned} \tag{1}
$$

where $K_E = pN_E$ and $K_I = pN_I$. The global input conductance of the neuron μ_G controls neurons' effective membrane time constant: τ_m:

$$
\begin{aligned}
&\mu_G(\nu_e,\nu_i) = \mu_{Ge} + \mu_{Gi} + g_L \\
&\tau_{\mathrm{m}}^{\mathrm{eff}}(\nu_e,\nu_i) = \frac{C_m}{\mu_G}
\end{aligned} \tag{2}
$$

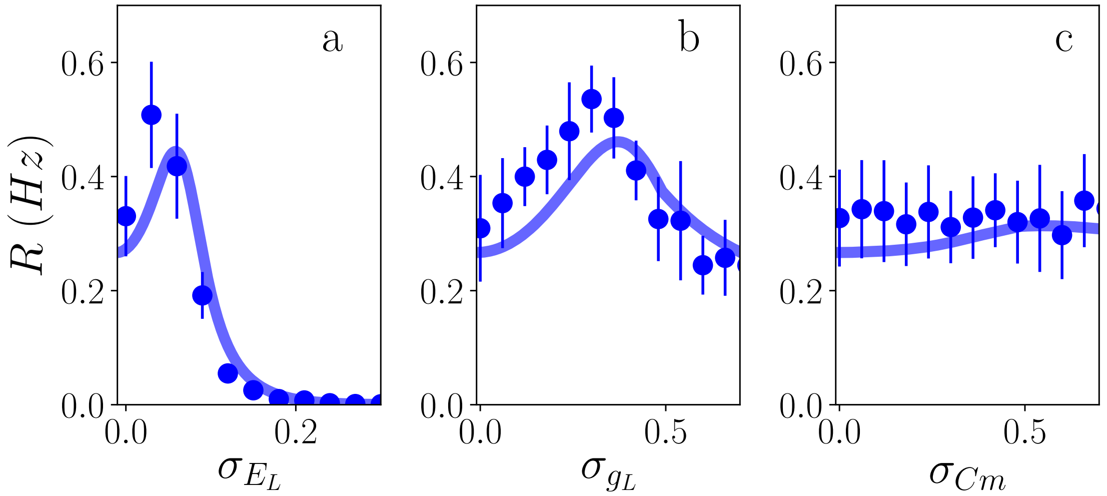

Figure 1. Responsiveness and cellular heterogeneity Responsiveness R in function of heterogeneity in inhibitory cell leakage reversal σ_{E_L} (panel a), leakage conductance σ_{g_L} (panel b) and membrane capacitance σ_{C_m} (panel c). Blue dots correspond to network simulations and error bars are estimated as the standard deviation over 20 different realisations. Continuous lines report the prediction based on the mean field model. In order to avoid boundary problems we have considered a higher value of mean g_L (with respect to Fig.1 of the main article), i.e. $\overline{g_L} = 40$nS. The average leakage reversal is $E_L^I = -63$mV ($E_L^E = -80$mV) for inhibitory (excitatory) cells. Input amplitude is $A = 1$Hz.

As a result, the mean voltage reads:

$$\mu_V(\nu_E, \nu_I, w) = \frac{\mu_{Ge} E_e + \mu_{Gi} E_i + g_L E_L - w}{\mu_G}. \tag{3}$$

The calculation of σ_V and of τ_V yields[3]:

$$\sigma_V(\nu_E, \nu_I) = \sqrt{\sum_s K_s \nu_s \frac{(U_s \cdot \tau_s)^2}{2\,(\tau_{\mathrm{m}}^{\mathrm{eff}} + \tau_s)}} \tag{4}$$

$$\tau_V(\nu_E, \nu_I) = \Big(\frac{\sum_s \left(K_s \nu_s (U_s \cdot \tau_s)^2 \right)}{\sum_s \left(K_s \nu_s (U_s \cdot \tau_s)^2 / (\tau_{\mathrm{m}}^{\mathrm{eff}} + \tau_s) \right)} \Big), \tag{5}$$

where we defined $U_s = \frac{Q_s}{\mu_G}(E_s - \mu_V)$.

From sub-threshold voltage moments to the output firing rate

Once calculated $(\mu_V, \sigma_V, \tau_V)$ as a function of (ν_E, ν_I, w) we evaluate the output firing rate of a neuron according to the following formula:

$$\nu_{out} = \frac{1}{2\,\tau_V} \cdot Erfc\left(\frac{V_{thre}^{eff} - \mu_V}{\sqrt{2}\,\sigma_V} \right). \tag{6}$$

Table 1. Fit parameters (expressed in mV)

Cell type	P_0	P_{μ_V}	P_{σ_V}	$P_{\tau_V^N}$	$P_{\mu_V^2}$	$P_{\sigma_V^2}$	$P_{(\tau_V^N)^2}$	$P_{\mu_V\sigma_V}$	$P_{\mu_V\tau_V^N}$	$P_{\sigma_V\tau_V^N}$
**E**	-53.1	7.87	-25.1	10.8	-2.4	-20.7	-20.7	13.5	-4.9	-8.4
**I**	-57.7	9.32	-35.9	22.1	-3.7	-27.8	-44.5	17.3	-1.2	-35.3

It has been shown, both theoretically and experimentally[1], that the voltage effective threshold V_{thre}^{eff} can be expressed as a function of $(\mu_V, \sigma_V, \tau_V)$. In particular, the phenomenological threshold was taken as a second order polynomial in the following way:

$$V_{thre}^{eff}(\mu_V, \sigma_V, \tau_V^N) = P_0 + \sum_{x \in \{\mu_V, \sigma_V, \tau_V^N\}} P_x \cdot \left(\frac{x - x^0}{\delta x^0}\right) + \sum_{x,y \in \{\mu_V, \sigma_V, \tau_V^N\}^2} P_{xy} \cdot \left(\frac{x - x^0}{\delta x^0}\right)\left(\frac{y - y^0}{\delta y^0}\right), \qquad (7)$$

where we introduced the adimensional quantity $\tau_V^N = \tau_V G_l / C_m$. We evaluated $\{P\}$ through a fit according to simulations on single neurons activity setting first $\mu_V^0 = -60$mV, $\sigma_V^0 = 0.004$mV, $(\tau_V^N)^0 = 0.5$, $\delta\mu_V^0 = 0.001$mV, $\delta\sigma_V^0 = 0.006$mV and $\delta(\tau_V^N)^0 = 1$. In table 1 we report the result of the fit.

3 Robustness of Responsivness

We report here a network simulation witht the sama parameters of Fig.2 of the main paper but for different Network realizations and initial conditions of the model variables. We can observe that, apart from finite size fluctuations, the Responsivness has a similar behavior in function of the heterogeneity levels (see Fig.2).

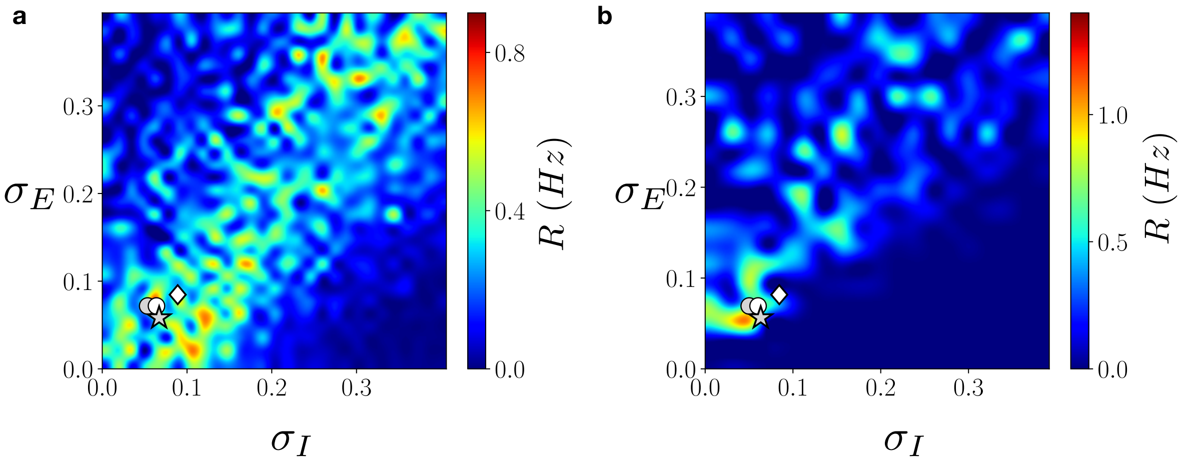

Figure 2. Responsiveness and cellular heterogeneity for different network realizations Responsiveness R in function of heterogeneity. Same simulations as in Fig.2 of the main paper for different Network realizations and initial conditions.

References

1. Zerlaut, Y. *et al.* Heterogeneous firing rate response of mouse layer v pyramidal neurons in the fluctuation-driven regime. *The J. physiology* **594**, 3791–3808 (2016).
2. Kuhn, A., Aertsen, A. & Rotter, S. Neuronal integration of synaptic input in the fluctuation-driven regime. *J. Neurosci.* **24**, 2345–2356 (2004).
3. Zerlaut, Y., Chemla, S., Chavane, F. & Destexhe, A. Modeling mesoscopic cortical dynamics using a mean-field model of conductance-based networks of adaptive exponential integrate-and-fire neurons. *J. computational neuroscience* **44**, 45–61 (2018).
